# Non-invasive brain stimulation combined with psychosocial intervention for depression: a systematic review and meta-analysis

**DOI:** 10.1186/s12888-022-03843-0

**Published:** 2022-04-19

**Authors:** Jiali He, Yiling Tang, Jingxia Lin, Guy Faulkner, Hector W. H. Tsang, Sunny H. W. Chan

**Affiliations:** 1 Department of Rehabilitation Sciences, The Hong Kong Polytechnic University, Hung Hom, Kowloon, Hong Kong; 2 School of Kinesiology, University of British Columbia, Vancouver, British Columbia Canada; 3 Mental Health Research Centre, The Hong Kong Polytechnic University, Hong Kong, SAR China; 4 School of Health and Social Wellbeing, University of the West of England, England, UK

**Keywords:** NIBS, Psychosocial intervention, Depression, Systematic review, Meta-analysis

## Abstract

**Objectives:**

This review investigates the efficacy and safety of non-invasive brain stimulation (NIBS) combined with psychosocial intervention on depressive symptoms.

**Materials and methods:**

We systematically searched five electronic databases from their inception to June 2021: PubMed, Embase, PsycINFO, Web of Science, and Medline. Randomized or non-randomized clinical trials in which NIBS plus psychosocial intervention was compared to control conditions in people with depressive symptoms were included.

**Results:**

A total of 17 eligible studies with 660 participants were included. The meta-analysis results showed that NIBS combined with psychosocial therapy had a positive effect on moderate to severe depression ([SMD = − 0.46, 95%CI (− 0.90, − 0.02), *I*^*2*^ = 73%, *p* < .01]), but did not significantly improve minimal to mild depression ([SMD = − 0.12, 95%CI (− 0.42, 0.18), *I*^*2*^ = 0%, *p* = .63]). Compared with NIBS alone, the combination treatment had a significantly greater effect in alleviating depressive symptoms ([SMD = − 0.84, 95%CI (− 1.25, − 0.42), *I*^*2*^ = 0%, *p* = .93]). However, our results suggested that the pooled effect size of ameliorating depression of NIBS plus psychosocial intervention had no significant difference compared with the combination of sham NIBS [SMD = − 0.12, 95%CI (− 0.31, 0.07), *I*^*2*^ = 0%, *p* = .60] and psychosocial intervention alone [SMD = − 0.97, 95%CI (− 2.32, 0.38), *I*^*2*^ = 72%, *p* = .01].

**Conclusion:**

NIBS when combined with psychosocial intervention has a significant positive effect in alleviating moderately to severely depressive symptoms. Further well-designed studies of NIBS combined with psychosocial intervention on depression should be carried out to consolidate the conclusions and explore the in-depth underlying mechanism.

**Supplementary Information:**

The online version contains supplementary material available at 10.1186/s12888-022-03843-0.

## Introduction

Depression is one of the most severe mental illnesses around the world affecting more than 264 million people globally [1]. A recent US study showed that the percentage of adults with depression has significantly increased from 8.7 to 14.4% during the COVID-19 outbreak [2]. At present, pharmacotherapy is still the first-line treatment for depression [3]. However, around 10 to 30% of people with depression experience treatment-resistant depression (TRD) during pharmacotherapy [4] and are less likely to remit on further trials after a few trials of antidepressant medication [5]. Given potential limitations of pharmacotherapy for some patients, more treatment options need to be considered to reduce the medical and financial burden of depression.

Non-invasive brain stimulation (NIBS) techniques are increasingly used to treat mental disorders. The most common NIBS techniques are transcranial direct current stimulation (tDCS) and repetitive transcranial magnetic stimulation (rTMS) [6, 7]. tDCS modulates cortical activities and excitability that are related to the symptoms of depression by applying weak electric direct currents over the frontoparietal network of the scalp [8, 9]. In contrast, rTMS is a more focal form of stimulation which has been approved to treat depression by the U.S. Food and Drug Administration (FDA) in 2008. It is delivered over the prefrontal cortex to induce a magnetic field to modulate the functional connectivity within and between two cortical networks which may alleviate depressive symptoms [10, 11].

Both techniques have been suggested to be effective and safe in treating depression in adults, but the effects of tDCS and rTMS vary according to multiple factors such as intensity, frequency, or pattern [9]. Furthermore, numerous studies have shown that the state of the targeted brain region has a great influence on the effects of NIBS [12, 13]. Considering that the effects are state-dependent, Sathappan et al. (2019) suggested that controlling the sustained neural activity in the targeted region and associated networks during NIBS stimulation may improve therapeutic outcomes and reduce inter-individual variability in response. Typically, NIBS techniques are often used in combination to produce therapeutic synergy in practice even though they are usually researched and developed as monotherapies [14].

Psychosocial intervention such as Cognitive Behavioral Therapy (CBT), Interpersonal Psychotherapy (IPT) have been developed to treat depression and present less safety concerns. Psychosocial intervention is defined as an intervention that includes interpersonal or informational activity, techniques, or strategies that focus on producing changes in biological, psychological, behavioral, cognitive, emotional, functional outcomes to improve well-being. Such intervention has been widely utilized as effective clinical treatment approaches for individuals with depression [15]. Importantly, each of the psychosocial intervention has been shown to be associated with promoting neural processing, for example, depressed individuals following IPT showed significant changes in the left temporal lobe, the right middle frontal gyrus (including the DLPFC), and left middle ACC metabolism [16, 17].

Expanding literature has demonstrated that the combination of NIBS with psychosocial therapies could potentially maximize the effects of NIBS or enhance the effects of the psychosocial therapy [14, 18]. For instance, a previous study suggested that combination of NIBS with other behavioral therapies has greater impact on increasing motor and speech functioning among stroke survivors [18]. Early in 2019, there was a systematic review that reviewed the combination of NIBS with cognitive intervention on neuropsychiatric illness. They identified five studies (3 with tDCS and 2 with rTMS) related to major depressive disorder treatment. However, the authors included different study designs in their review and did not conduct a meta-analysis [14].

To build on this work, we conducted a systematic review and meta-analysis assessing the efficacy of combining active NIBS with various psychosocial intervention when compared with the combination of sham NIBS, NIBS alone or psychosocial intervention alone. We hypothesize that NIBS in combination with psychosocial intervention could potentially have a greater impact on depression symptoms than either technique individually. NIBS stimulation parameters, drop-out rate, and adverse events were also assessed to guide future clinical practice in the management of depression among adults, especially for high-severity depression.

## Materials and methods

This systematic review and meta-analysis was conducted following the PRISMA guidelines 2020 [19]. A PRISMA checklist was provided in Supplement 1. The registration number in PROSPERO is: CRD42021273363.

### Search strategy

Five electric databases (PubMed, Embase, PsycINFO, Web of Science, Medline) were searched for records in English from inception to June 20, 2021. Searches were conducted using combinations of the following keywords: “non-invasive brain stimulation” OR “transcranial direct current stimulation” OR “tDCS” OR “transcranial magnetic stimulation” OR “TMS” OR “repetitive transcranial magnetic stimulation” OR “rTMS” AND “depress*” OR “mental health” OR “mental disorder*” OR “psychiatric disorder*” OR “mood disorder*” OR “bipolar disorder*”. Detailed search strategies were presented as the Supplement 2. The reference lists of included studies were also searched to identify potentially eligible articles.

### Eligibility criteria

The selection criteria were based on the Population, Intervention, Comparison, Outcome, Settings (PICOS) framework [20]. Studies that met the following criteria were included in this review:

- Participants: Adults (≥18 years old) with minimal to severe depressive symptoms determined based on validated depression scales.
- Interventions: Intervention involved one of the NIBS techniques (rTMS or tDCS) combined with a psychosocial intervention. The psychosocial intervention could be performed in any face-to-face format (individually or in groups). Any studies including pharmacotherapy were not eligible.
- Comparison: Comparisons between NIBS combined with psychosocial therapy and sham NIBS combined with psychosocial therapy, NIBS-alone, or psychosocial therapy alone were all included.
- Outcomes: Depressive symptoms were measured and reported in original studies as the primary or secondary outcome with clinically diagnostic scales or standardized self-reported scales. If detailed data (mean and SD) of depression were insufficiently reported and could not be retrieved from the authors, the study was removed.
- Study designs: Randomized controlled trails (RCTs) and non-RCTs were included.

### Study selection

Two review authors (JH and YT) independently screened the titles and the abstracts of the potentially eligible studies on Covidence systematic review software [21]. In addition, the full text of identified studies was retrieved and evaluated independently by the same two authors. Any disagreement related to the study design and the final decision of including studies between review authors was resolved in a consensus meeting.

### Data extraction

Two authors (JH and YT) individually extracted the data from included articles. Conflicts were resolved by further discussion. The following general characteristics were collected from included studies: authors and year of publication, sample size, participant characteristics, methodological design, intervention protocols, details of control groups, depressive outcomes, drop-out rate, and adverse events.

### Quality assessment

Two reviewers (JH and YT) independently assessed the quality of each included publication using the revised Cochrane Risk of Bias assessment tool (ROB 2.0) for RCTs [22]. RoB 2.0 consists of five following domains: 1) bias arising from the randomization process; 2) bias due to deviations from the intended interventions; 3) bias due to missing outcome data; 4) bias in measurement of the outcome; and 5) bias in selection of the reported result.

### Quantitative analysis

All statistical analyses were conducted using RStudio statistical software (Boston, MA, USA). The meta-analysis of the pooled standardized mean difference (SMD) and the pooled standard deviation (SD) were calculated for each comparison on the basis of random effects model. The pooled effect size was estimated with Hedges’ g instead of Cohen’s d, which is considered preferred to correct for small sample size bias [23]. The value of Hedges’ g can be interpreted similarly as the standard Cohen’s d, i.e. the value of 0.2-0.5, 0.5-0.8, > 0.8 represents a small, medium and large effect size, respectively [24]. The median and range of the SMD were reported as well. *I*^*2*^ statistic was used to analyze heterogeneity for the meta-analyses. As the power of *I*^*2*^ test is low when only including a small number of studies or with small sample sizes, *p*-value ≤ .10 was considered as reflecting significant heterogeneity [25]. The Knapp-Hartung adjustments were applied to calculate the confidence interval (CI) around the pooled effect sizes to control the risk of false positive. Random-effects univariate meta-regression was also used to assess heterogeneity with the following covariates when comparing NIBS combined with psychosocial intervention with sham NIBS plus psychosocial intervention: age, clinical condition (MDD vs. other disease), total sessions of NIBS and psychosicial intervention protocols, stimulation sequence of NIBS, and stimulation site of NIBS (prefrontal cortex, frontal coetex, and central cortex). For tDCS, we meta-regressed current density (1.0 mA vs. 2.0 mA). For rTMS, no meta-regressions were performed due to the limited number of studies. According to guidance, meta-regression should generally not be performed when there are less than 10 studies in the meta-analysis [25]. Sensitivity analysis was used to investigate the stability of the results through calculating the resulting effect size by removing each individual study, including one study with high risk of bias according to RoB 2.0. Begg’s funnel plot and Egger’s regression test were used to investigate publication bias [26]. Finally, separate meta-analyses were performed to investigate the effect of NIBS combined with psychosocial intervention on the improvement of depression compared to the control group (sham NIBS combined with psychosocial intervention, NIBS alone and psychosocial intervention alone).

## Results

### Selection of studies

**Fig. 1:**
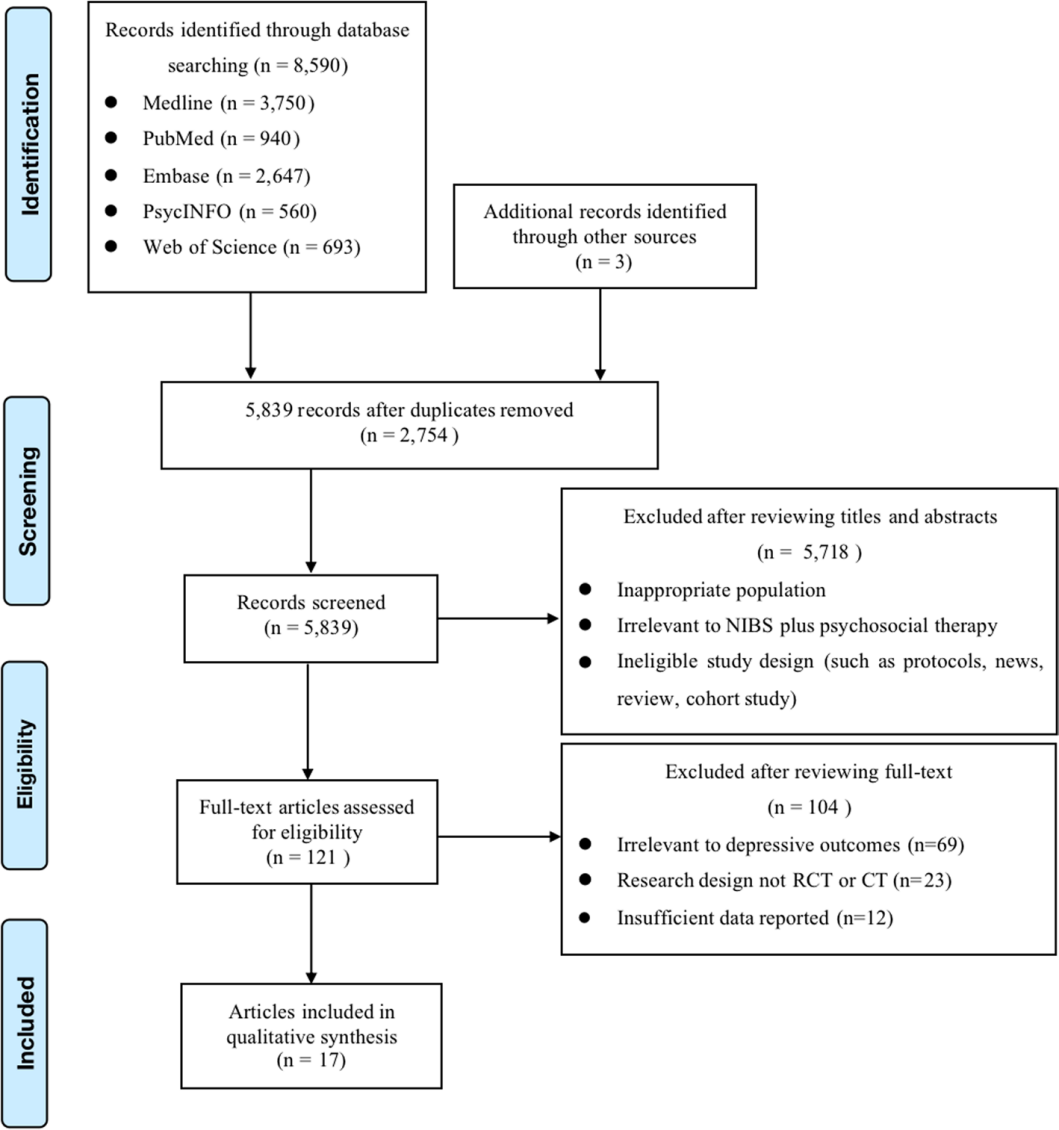
PRISMA flow diagram of search results

Figure 1 depicts the PRISMA flow diagram. The search criteria initially yielded 8590 articles from the databases based on the proposed keywords and manually added additional 3 articles through reviewing reference lists of retrieved articles and review studies. 2754 duplicates were removed by Covidence and 5718 articles were excluded after screening the title and abstract. One hundred and twenty-one were potentially relevant to our systematic review on the basis of the eligibility criteria. After full text evaluation, 104 articles were excluded for the following reasons: irrelevant outcomes (*n* = 69), wrong study design (*n* = 23), and insufficient data reported (*n* = 12). Finally, 17 articles were included in the meta-analysis.

### Study characteristics

The main features of the included studies are summarized in Table 1. The 17 articles involved 660 participants with sample sizes varying from 9 to 100. Three hundred and twelve participants were assigned to intervention groups and 348 participants to control conditions. Eight studies (47.06%) recruited participants diagnosed with psychiatric disorders. In contrast, the rest of studies recruited participants with physical illnesses comorbid with depression. The severity of participant’s depression in each study was classified based on the criteria of different diagnostic tools of the baseline assessment. The definitions of different severity of depression for each diagnostic tool have been summarized in Supplement 3 [44–48]. Finally, the subjects in 5 studies (29.41%) were considered as minimal to mild depression [30, 33–35, 41]. The remaining 12 studies (70.59%) included samples indicative of moderate to severe depression.

**Table 1:** Overview of included studies

Author, year	NIBS technique	Psychosocial intervention	Target population	Sample size (I/C)	Age Mean (SD)	Gender (F/M)	Duration Mean (SD)	Depressive outcome
Brunoni et al., 2014 [27]	Anodal tDCS	Cognitive control therapy	Major depressive disorder (MDD)	37(20/17)	IG: 46.1(10.4) CG: 41.5(10.6)	IG: 7/13 CG: 4/13	IG: 17.4(15.8) M CG: 9.2(9.2) M	HDRS-21 BDI
Guinot et al., 2021 [28]	HF-rTMS	Exercise training	Fibromyalgia (FM)	39(20/19)	IG: 46.5(10.4) CG: 42.8(8.8)	IG: 18/0 CG: 15/4	IG: 11.2(10.9) Y CG: 9.2(9.6) Y	BDI
Khayyer et al., 2018 [29]	Anodal tDCS	Positive psychotherapy	MDD	9(3/3/3)	45	N/A	N/A	HDRS
Lagueux et al., 2018 [30]	Anodal tDCS	Graded motor imagery	Complex regional pain syndrome	22(11/11)	IG: 41(9) CG: 53(10)	IG: 8/3 CG: 6/5	IG: 36(26) M CG: 37(26) M	BDI-II
Lee and Kim, 2018 [31]	LF-rTMS	Neurodevelopmental therapy	Traumatic brain injury	13(7/6)	IG: 42.42(11.32) CG: 41.33(11.02)	IG: 2/5 CG: 2/4	IG: 3.85(1.67) M CG: 3.88(1.94) M	MADRS
Li et al., 2021 [32]	HF-rTMS	Occupational therapy	Morphine dependence	100(50/50)	IG: 33.8 (7.5) CG: 36.2 (8.0)	IG: 14/36 CG: 11/39	IG: 6.8(3.6) Y CG: 7.1(3.3) Y	SDS
Manenti et al., 2016 [33]	Anodal tDCS	Physical therapy	Parkinson	20(10/10)	IG: 69.0 (9.1) CG: 69.1 (5.6)	IG: 6/4 CG: 3/7	IG: 7.1(3.6) Y CG: 7.8(4.2) Y	BDI-II
Manenti et al., 2018 [34]	Anodal tDCS	Computerized cognitive training	Parkinson	22(11/11)	IG: 65.5(6.4) CG: 63.8(7.1)	IG: 6/5 CG: 4/7	IG: 6.2(3.9) Y CG: 7.6(3.4) Y	BDI-II
Martin et al., 2019 [35]	Anodal tDCS	Cognitive training	Mild cognitive impairment	68(33/35)	IG: 71.8(6.39) CG: 71.6(6.35)	IG: 20/13 CG: 25/10	N/A	MADRS
Mendonca et al., 2016 [36]	Anodal tDCS	Aerobic exercise	FM	45(15/15/15)	IG: 44.5(14) CG1: 48.0(11.8) CG2: 49.9(10.6)	IG: 14/1 CG1: 15/0 CG2: 15/0	IG: 140.6(72.2) M CG1:149.3(111.1) M CG2: 126.6(100.2) M	BDI
Nasiri et al., 2020 [37]	Anodal tDCS	Unified protocol treatment	Generalized anxiety disorder and comorbid depression	47 (15/15/17)	IG: 20.23(2.89) CG1: 21.53(3.56) CG2: 20.53(2.53)	IG: 10/3 CG1: 11/4 CG2: 11/4	N/A	BDI-II
Nord et al., 2019 [38]	Anodal tDCS	Cognitive behavioral therapy (CBT)	MDD	39(20/19)	IG: 35.60(12.91) CG: 31.05(8.17)	IG:9/11 CG: 11/8	N/A	BDI HAM-D
Riberto et al., 2011 [39]	Anodal tDCS	Multidisciplinary rehabilitation program	FM	23(11/12)	IG: 58.3(12.1) CG: 52.4(11.5)	N/A	IG: 141.63 (184.11) M CG1: 84.11 (102.09) M CG2: 37.33 (39.12) M	BDI HDRS
Segrave et al., 2014 [40]	Anodal tDCS	Cognitive control training	MDD	27(9/9/9)	IG: 42.6(18.32) CG1: 45.0(10.15) CG2: 33.8(12.96)	IG: 2/7 CG1: 4/5 CG2: 4/5	N/A	MADRS BDI-II
Sharma et al., 2020 [41]	LF-rTMS	Conventional physical therapy	Ischemic stroke	100(50/50)	IG: 54.85(13.39) CG: 52.89(14.95)	IG: 14/33 CG: 15/34	N/A	HAM-D
Van Noppen et al., 2020 [42]	Anodal tDCS	Behavioral therapy	Burnout	16(8/8)	IG: 42.5(5.5) CG: 47.4(5.3)		N/A IG: 31.31(8.17) M CG: 10(9.63) M	BDI
Vanderhasselt et al., 2015 [43]	Anodal tDCS	Neurocognitive training	MDD	33(19/14)	IG: 42.26(10.67) CG: 41.00(11.54)	IG: 13/6 CG: 11/3	NA	HAM-D BDI-II

BDI: Beck Depression Inventory; BDI-II: Beck Depression Inventory-II; HAM-D: Hamilton Rating Scale for Depression; HDRS-21: Hamilton Depression Rating Score, 21-items; MADRS: Montgomery-Asberg Depression Rating Scale; SDS: Self-rating Depression Scale

The detailed protocols of the NIBS and psychosocial intervention are presented in Supplement 4. Nearly all anodal tDCS protocols had a current intensity of 2.0 mA to stimulate the cortex, except for 3 studies (17.65%) which used 1.0 mA and 1.5 mA respectively. The stimulated time of tDCS varied from 20 to 30 min and the total number of sessions ranged from 5 to 15. For the rTMS protocols, low-frequency rTMS (1 Hz) were applied in 2 studies (11.76%) to inhibit the corresponding cortical areas, while 2 studies (11.76%) used high-frequency rTMS (above 5 Hz) to excite cerebral cortex. The time of each stimulation lasted from 20 to 30 min, and the total number of sessions ranged from 10 to 40. The psychosocial intervention in all eligible studies including psychotherapy, physical therapy, and occupational therapy. The duration of psychosocial intervention in all trials lasted from 10 min to 60 min and the total number of sessions ranged from 3 to 108.

Four studies (23.53%) compared the effect of the combination treatment with NIBS alone or psychosocial intervention alone. Thirteen studies (76.47%) analyzed the efficacy of NIBS plus psychosocial intervention versus a control group of the combination with sham NIBS. Furthermore, 4 studies (23.53%) assessed the effect of rTMS combined with psychosocial intervention and the remaining 13 studies (76.47%) studies assessed the effect of tDCS combined with psychosocial intervention. Importantly, the onset of NIBS was applied at the same time, before and right after the combined psychosocial intervention in 4 (23.53%), 6 (35.29%) and 4 studies (23.53%) respectively. But 3 included studies (17.65%) did not describe the treatment consequence of NIBS stimulation and psychosocial intervention.

### Risk of bias within studies

**Fig. 2:**
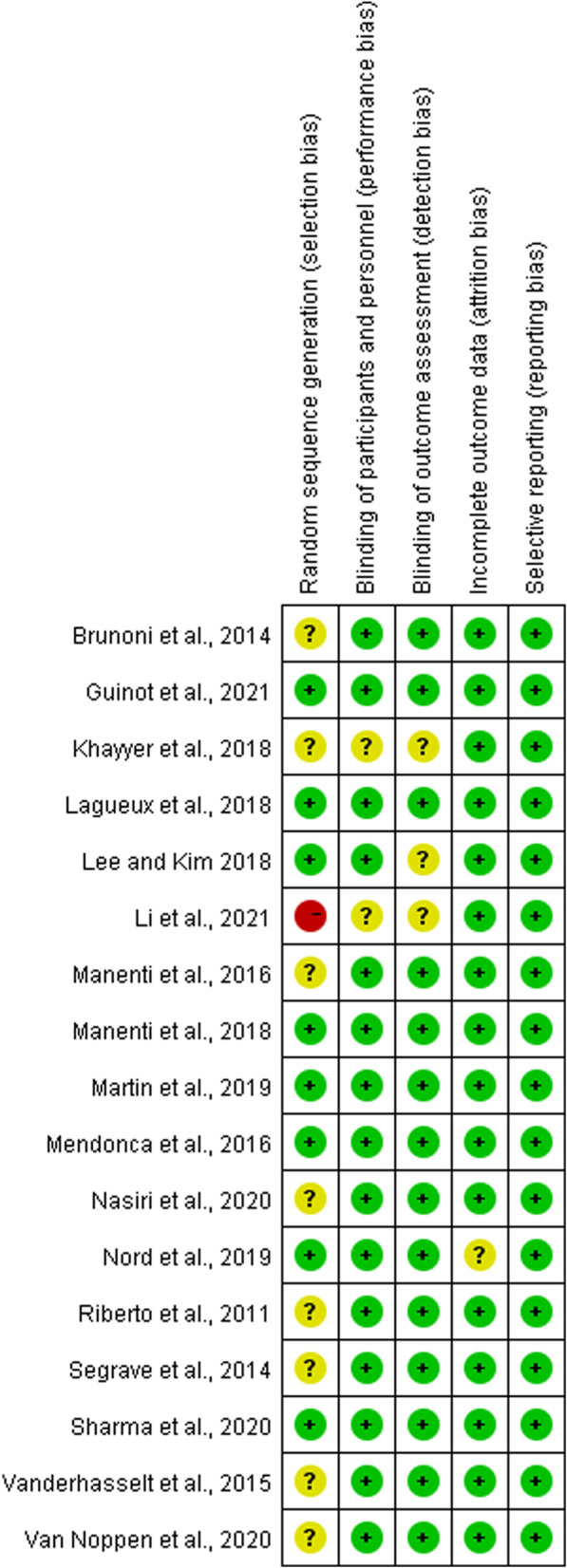
The risk of bias graph of included studies

**Fig. 3:**
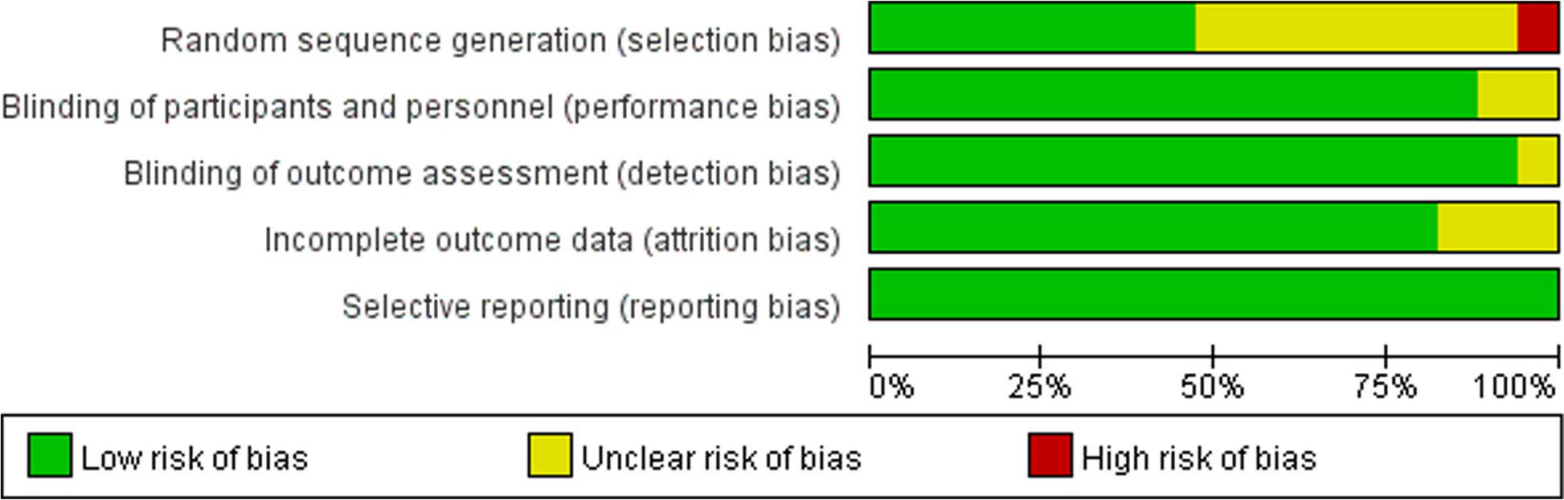
The risk of bias summary of included studies

The risk of bias for all eligible studies was assessed and is presented in the risk of bias graph (Fig. 2) and the risk of bias summary (Fig. 3). All studies were described as randomized except one study whose selection bias was considered as high. Of these studies, 8 studies (47.06%) only reported random grouping but did not mention allocation concealment in details and were consequently judged to be unclear in this domain. The performance bias of 13 studies (76.47%) was determined as low since these studies were double-blind for both participants and assessors. Only one study (5.89%) reported blinding of participants only. Three studies (17.65%) did not report blinding, and these were considered to have a potentially high risk of performance bias and detection bias. Regarding incomplete outcome data, only one study (5.89%) described the post-randomization drop-outs, and the participants were excluded from the analysis. Therefore, this study was determined as unclear risk of attrition rate. All 17 studies (100%) clearly reported the important outcomes (depressive outcomes) and were rated as low risk of selective outcome reporting bias.

### Synthesis of results

#### Efficacy of combined interventions on different depressive levels

**Fig. 4:**
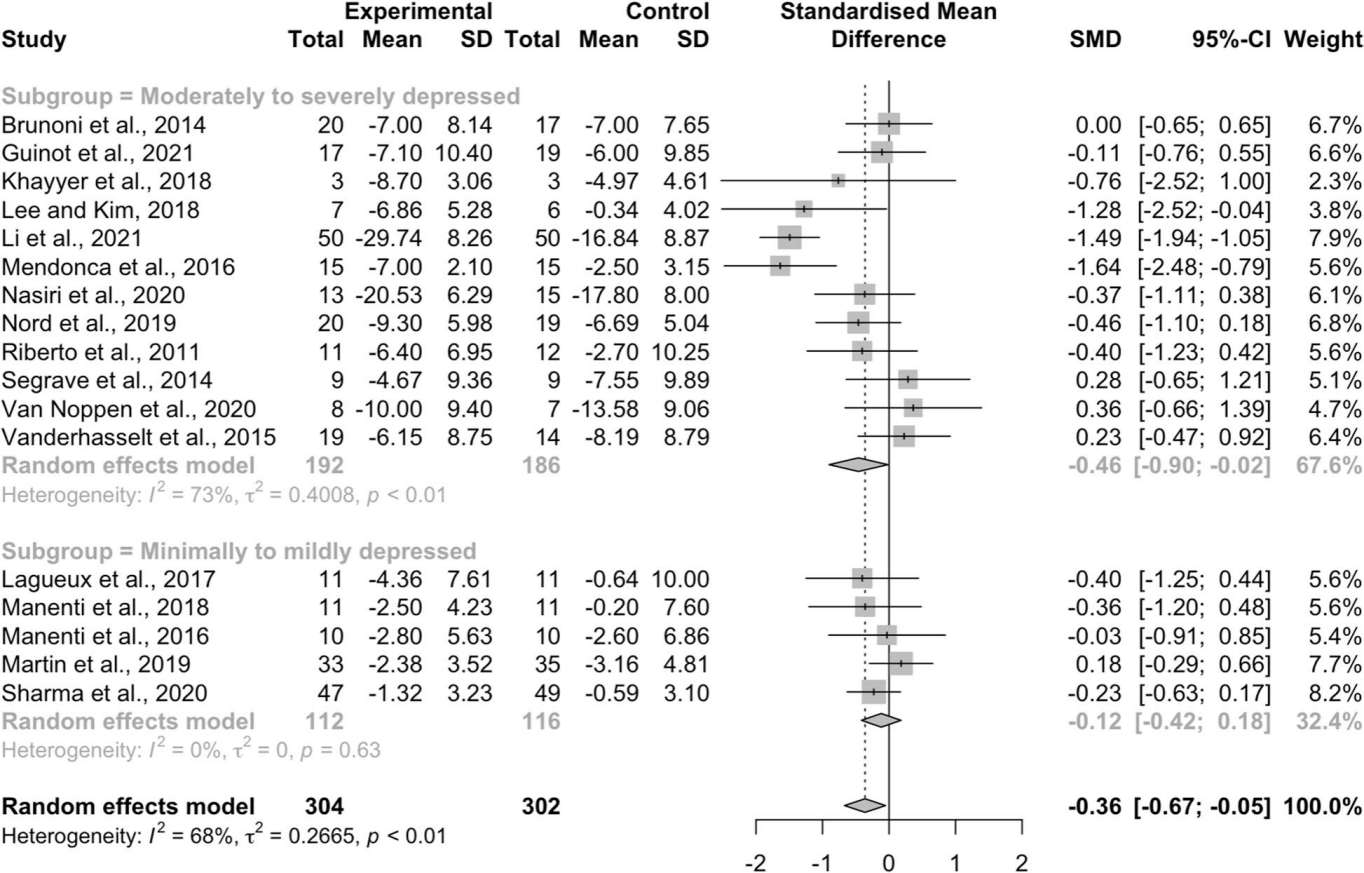
Effects of NIBS plus psychosocial intervention for different severity of depression

**Fig. 5:**
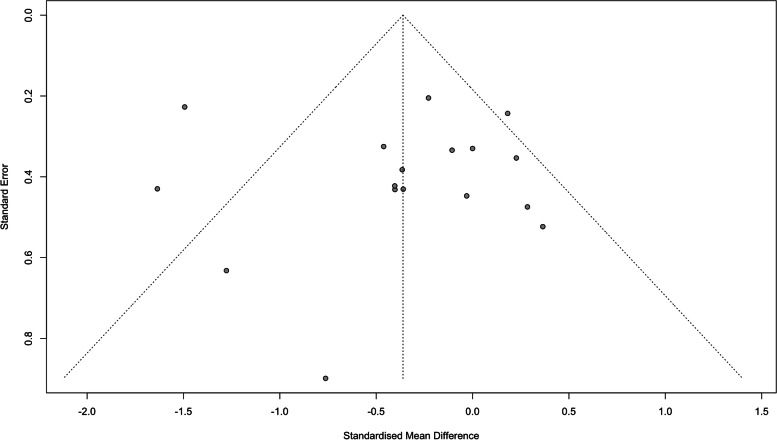
Funnel plots of eligible studies

Of the 17 included studies, the pooled meta-analysis results reflected that NIBS combined with psychosocial intervention in treatment depression yielded a small effect on depression [SMD = − 0.36, 95%CI (− 0.67, − 0.05), *I*^*2*^ = 68%, *p* < .01], with an effect size median of − 0.36 ranging from − 1.64 to 0.36 (Fig. 4). Visual inspection of the Begg’s funnel plot (Fig. 5) and Egger’s regression test results (*p* = .82) suggests there is low risk of publication bias. Furthermore, sensitivity analyses showed that no particular study substantially changed the pooled effect, which varied from − 0.36 to − 0.24 [95%CI (− 0.47, − 0.01), *I*^*2*^ = 34%, *p* = .09) after excluding one study with high risk of bias [32].

Based on severity levels of depression, all studies were divided into two subgroups: moderately to severely depressed and minimally to mildly depressed. Twelve studies (70.59%) evaluated the effects of combined interventions for moderate to severe depression and the subgroup analysis results demonstrated that NIBS plus psychosocial intervention elicited a notable improvement in depression, with a small to moderate effect [SMD = − 0.46, 95%CI (− 0.90, − 0.02), *I*^*2*^ = 73%, *p* < .01], with an effect size median of − 0.39 ranging from − 1.64 to 0.36. Five studies (29.41%) assessed the effects of the combined interventions for minimal to mild depression, with results indicating that compared with control groups, NIBS combined with psychosocial intervention did not significantly alleviate depressive symptoms [SMD = − 0.12, 95%CI (− 0.42, 0.18), *I*^*2*^ = 0%, *p* = .63]. The median effect size was − 0.23 and ranged from − 0.40 to 0.18.

#### Efficacy of combined interventions compared with various control groups

Based on the different types of control group, the studies were categorized into comparisons of:

1. NIBS plus psychosocial intervention versus sham NIBS plus psychosocial intervention.
2. NIBS plus psychosocial intervention versus NIBS alone.
3. NIBS plus psychosocial intervention versus psychosocial intervention alone.

**Fig. 6:**
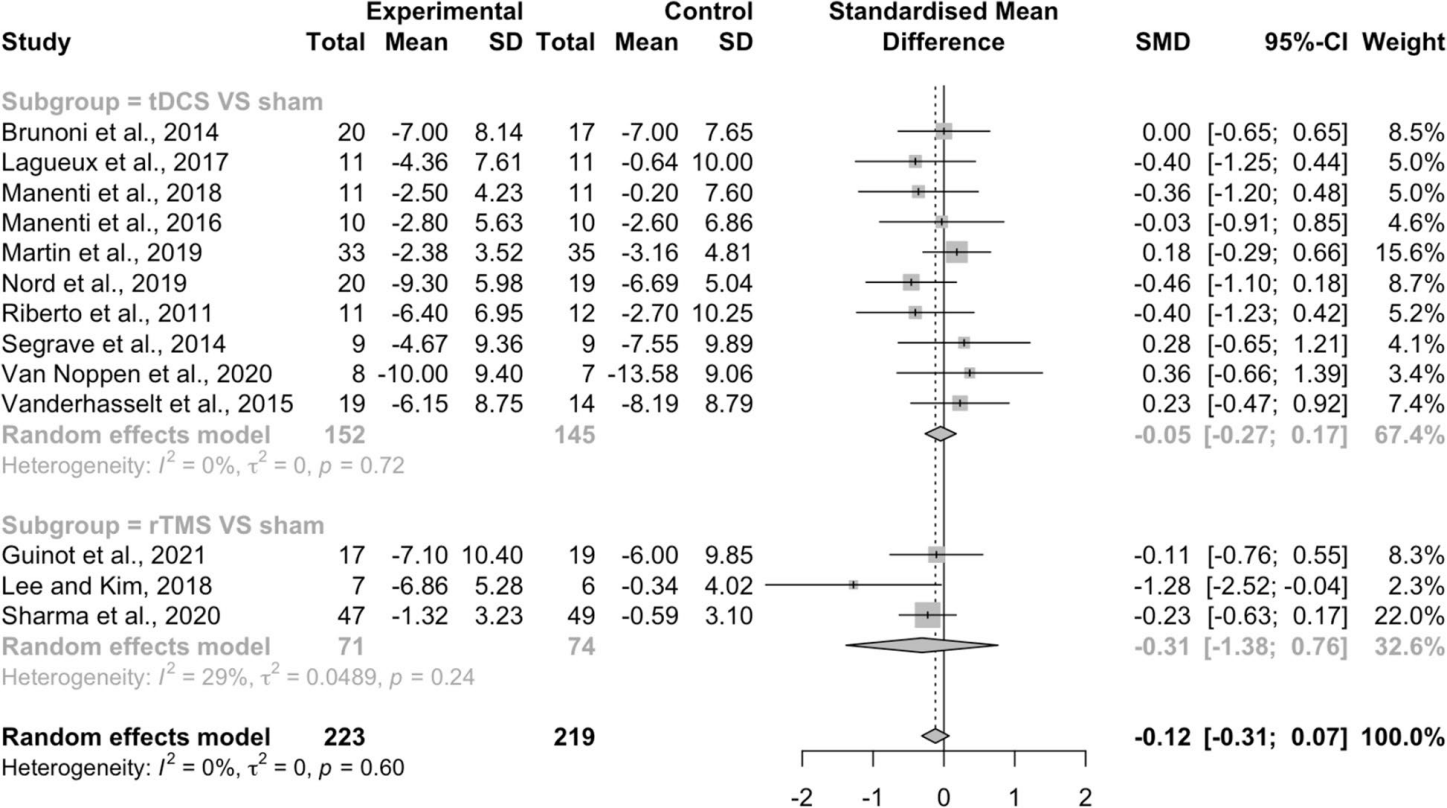
Effects of NIBS plus psychosocial intervention compared to the combination of sham NIBS

Thirteen studies (76.47%) analyzed the effects of NIBS combined with psychosocial intervention compared to the combination of sham NIBS with psychosocial intervention. The pooled results of the meta-analysis showed that NIBS plus psychosocial intervention had no significant effect on depression compared with sham NIBS plus psychosocial intervention [SMD = − 0.12, 95%CI (− 0.31, 0.07), *I*^*2*^ = 0%, *p* = .60], with a median effect size of − 0.11 and ranging from − 1.28 to 0.36 (Fig. 6). Regarding the type of NIBS protocol, the subgroup meta-analysis demonstrated that tDCS combined with psychosocial intervention did not improve depressive symptoms when comparing with the combination of sham tDCS [SMD = − 0.05, 95%CI (− 0.27, 0.17), *I*^*2*^ = 0%, *p* = .72], with a median effect size of − 0.02 and ranging from − 0.46 to 0.36. In addition, rTMS combined with psychosocial intervention had no significant effect in alleviating depressive symptoms when compared with sham rTMS plus psychosocial intervention [SMD = − 0.31, 95%CI (− 1.38, 0.76), *I*^*2*^ = 28%, *p* = .25], with the median effect size of − 0.23 and ranging from − 0.11 to − 1.28.

**Fig. 7:**
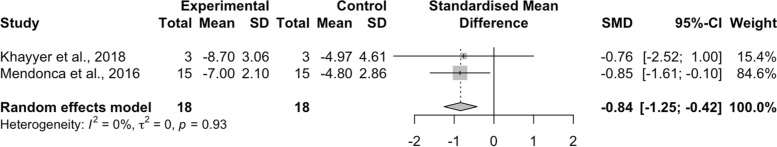
Effects of NIBS plus psychosocial intervention compared to NIBS alone control group

Two studies (11.76%) evaluated the effects of NIBS combined with psychosocial intervention compared to NIBS alone. The pooled results of the meta-analysis showed that the combined treatment had a large, significant effect in reducing depression [SMD = − 0.84, 95%CI (− 1.25, − 0.42), *I*^*2*^ = 0%, *p* = .93], with a median effect size of − 0.81 ranging from − 0.76 to − 0.85 (Fig. 7).

**Fig. 8:**
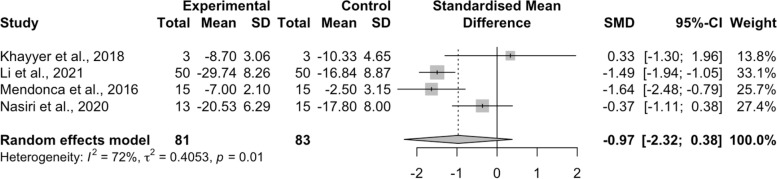
Effects of NIBS plus psychosocial intervention compared to psychosocial intervention alone control group

Four studies (23.53%) assessed the combination compared to psychosocial intervention alone. The pooled results of the meta-analysis showed that NIBS plus psychosocial intervention had no significant effect in reducing depression compared to the control group [SMD = − 0.97, 95%CI (− 2.32, 0.38), *I*^*2*^ = 72%, *p* = .01], with a median effect size of − 0.93 ranging from − 1.64 to 0.33 (Fig. 8).

#### Meta-regression

In order to evaluate the impact of various study characteristics on the study estimates of SMD, we also performed the univariate meta-regression analyses of the combined interventions for depression (Table 2). Our results suggested that the stimulation site was significantly associated with Hedges’ g effect sizes of the combination treatment, with greater effects in the frontal cortex than the central cortex (β = 0.74, *p* = .02). However, the results indicated that effects of the combination treatment were not significantly influenced by other covariates including age, clinical condition, total sessions of NIBS and psychosocial intervention protocols, stimulation sequence of NIBS, and current density of tDCS.

**Table 2:** Summary of univariate meta-regression analyses

Covariate	Coefficient (E)	95%CI		R2 (%) p-Value	
		Lower	Upper		
Continuous					
Participant’s age, year	0.01 (0.01)	-0.01	0.04	9.95	.24
Session of NIBS, times	0.10 (0.05)	0.00	0.20	20.83	.06
Session of psychosocial intervention, times	0.00 (0.00)	-0.01	0.01	0.00	.98
Density of tDCS, mA	0.26 (0.39)	-0.60	1.12	0.00	.67
Categorical					
Clinical condition					
MDD			Referent		
Other disease	-0.52 (0.32)	-1.19	0.16	9.28	.12
Stimulation sequence					
NIBS first			Referent		
NIBS last	-0.08 (0.21)	-0.55	0.38	0.00	.70
Simultaneous	0.26 (0.20)	-0.17	0.69	0.00	.21
Stimulation site					
Central			Referent		
Frontal	0.74 (0.30)	0.11	1.36	91.57	.02
Prefrontal	0.45 (0.49)	-0.62	1.51	91.57	.38

#### Drop-out rate

Six of the 17 studies (35.29%) reported the drop-out rate, and the mean drop-out rate was 5.05% ranging from 0.00 to 37.84% [27, 41]. For the intervention group, the mean drop-out rate was 2.16% ranging from 0.00 to 18.91% [27, 38]. For the control group of sham NIBS plus psychosocial intervention, the mean drop-out rate was 3.45% ranging from 0.00 to 18.91% [27, 41]. None studies (0.00%) reported the drop-out rate in NIBS alone group and only one studies (5.88%) mentioned a drop-out rate of 4.26% in psychosocial intervention alone group [37].

#### Main adverse events

Six studies (35.29%) reported numerical data about adverse events [30, 35, 36, 38, 41, 42]. One study reported that one participant developed seizure after 4 sessions of rTMS stimulation [41]. Common side effects were reported in the remaining studies including headache, scalp pain, skin redness, and itching in either NIBS or sham NIBS intervention group [30, 35, 36, 38, 42].

## Discussion

To the best of our knowledge, this is the first systematic review and meta-analysis evaluating the effects of NIBS combined with psychosocial intervention for people with depression. Seventeen studies with 660 participants were involved in this review. Our meta-analysis showed that NIBS combined with psychosocial intervention was effective in alleviating moderate to severe depression but not among individuals with minimally to mildly depressive symptoms. Furthermore, a larger effect size of combined interventions on depression was found when comparing with either NIBS alone. However, our results indicated that NIBS combined with psychosocial intervention had no specific enhancing effects on depressive symptoms compared to the combination of sham NIBS or psychosocial intervention alone.

In the past decade, neuroimaging studies have provided insights into the alterations in brain structure of individuals with depression to explore alternative and complementary treatments. For example, reduction of grey matter volume in several brain areas including anterior cingulate cortex (ACC), dorsolateral prefrontal cortex (DLPFC), and the hippocampus [49]. Individuals with depression may also have impaired coordinated activity in several cortical regions, such as network hyper-connectivity between ACC and PFC [50, 51]. Non-invasive neuromodulation, including tDCS and rTMS, have been demonstrated to activate or inhibit the excitability of the corresponding cortical regions, and modulate the functional connectivity of brain areas [52]. In addition, several neuroimaging studies have shown that psychosocial intervention can promote neural processing to improve neuropsychiatric disorders, for example, cognitive control training can modulate brain activity and decrease functional connectivity between various cortex regions [53, 54]. Given that brain activity can be regulated through both NIBS and psychosocial therapies and based on meta-regression results that the effect of the combination treatment on depressive improvement was not influenced by the simulation site of tDCS, we can eventually conceptualize the combined intervention as the modulation of overlapping neural circuits that occurs through independent but synergistic mechanisms. Considered together, the combined intervention could be suggested to apply in individuals with high-severity depression or difficult-to-treat depression, or the groups who fail to benefit adequately from currently standard clinical therapies.

For the individuals with minimal to mild depression, however, the combined intervention had no beneficial effects compared with controlled interventions possibly due to the significant effects of NIBS alone and psychosocial intervention alone for patient with less severe depression. Previous systematic reviews and meta-analyses have reported that NIBS and psychosocial intervention are effective to significantly accelerate improvements in depression, with a notably large effect size of 1.14 [6] and a medium effect size of 0.62 separately [55]. Our results demonstrated that the combination had a significant large effect of 0.84 on depressive symptoms compared with NIBS alone, suggesting that the combined intervention is a promising approach to maximize the benefits to be gained from NIBS intervention alone. In addition, we found that NIBS stimulation of the frontal cortex was statistically superior to stimulation of the central cortex when combined with psychosocial intervention. The findings are partially consistent with the current stimulation targets for depressive disorders [56]. Interestingly, other covariates did not find to manifest any statistical significances in our meta-regression model. But it could possibly be attributed to the limited number of each covariate, which resulting in insufficiently statistical power for difference detection.

However, compared with psychosocial intervention alone, no significant difference in reducing depression in the combination intervention group was found. In contrast, a recent scoping review showed that NIBS combined with CBT was significantly associated with changes in depressive symptoms [14]. However, the limited number of studies with each type of psychosocial interventions making it difficult to conduct subgroup analyses to evaluate whether NIBS plus specific type of psychosocial intervention is more effective than NIBS plus others. Therefore, exploring the effectiveness of some specific psychosocial interventions in combination with NIBS for the treatment of depression is the priority suggested for future research.

In addition, the effects of active NIBS plus psychosocial intervention were no different from those studies using sham NIBS. This is in line with the high rates of placebo effects found in previous systematic reviews for depression. In fact, several meta-analyses have already demonstrated that the placebo effect plays an important role in both tDCS [Hedges’ g = 1.09, 95% CI (0.8, 1.38)] and rTMS trials [Hedges’ g = 0.80, 95% CI (0.65, 0.95)] in depression [57, 58]. Of all the studies, only Segrave et al., (2014) compared three intervention arms that included sham-controlled groups (active tDCS combined, sham tDCS combined, sham CCT combined) without NIBS-alone and a psychosocial intervention alone control group. Other studies were all two-arm controlled trials comparing the combined treatment to sham NIBS combined with psychosocial therapies. Therefore, it is important for further RCTs to compare active combined, sham combined, NIBS-alone, and psychosocial intervention alone to more accurately evaluate the potential for NIBS and psychosocial intervention acting synergistically.

The drop-out rate was similar to previous studies of NIBS in adults with depression in both clinical and community settings [59, 60]. In terms of the safety of NIBS combined with psychosocial intervention, almost all studies suggested that it is a safe treatment with a few common and tolerable side effects, such as post-stimulation headache. However, one study reported that one participant experienced seizure after active rTMS stimulation [41]. In order to reduce such side effects happening, the pre-rTMS treatment evaluation is recommended to determine the health status of the individual. In addition, a TMS procedure needs to be performed to correctly establish the optimal site of motor responses and individual motor thresholds to minimize adverse effects [11].

Several limitations should be considered when interpreting this systematic review and meta-analysis. One important limitation is the moderate to high level heterogeneity of the combination intervention observed in our meta-analysis, which potentially limits interpretation. The included studies used different NIBS protocols and the definition of psychosocial intervention was extremely broad, but the limited number of eligible studies did not allow us to fully assess how these potential factors played a role in the heterogeneity. Also, the included studies differ substantially in clinical characteristics of participants, making comparisons difficult. Another point is that the small number of included studies with a small sample size could possibly decrease the power to evaluate the effect of the combination treatment when comparing with NIBS alone and psychosocial intervention alone. Altogether, these limitations could possibly affect the evidence grade of meta-analysis.

## Conclusion

This systematic review and meta-analysis found emerging evidence to support the enhanced effects of NIBS in combination with psychosocial intervention for individuals experiencing depression. Further multi-conditions and high methodological quality trials are required to explore the synergistic effects and in-depth underlying mechanisms of the combination of NIBS and psychosocial intervention. In addition, further research should look at a more focused definition of evidence-based psychosocial intervention as a comparison to provide robust evidence for the clinical management of depression. Importantly, the health status of participants and the appropriate stimulation parameters should be evaluated and determined prior to initiating NIBS stimulation.

## Supplementary Information

**Additional file 1..**

**Additional file 2..**

**Additional file 3..**

**Additional file 4..**

## Data Availability

All data generated or analysed during this study are included in this published article and its supplement 4.
