# Supplementary material for: Non-invasive brain stimulation combined with psychosocial intervention for depression: a systematic review and meta-analysis
Source: BMC Psychiatry. 2022 Apr 19;22:273. doi: 10.1186/s12888-022-03843-0 (PMC9016381; doi:10.1186/s12888-022-03843-0)
Supplement: Supplementary file 2 — Additional file 2.. [file 12888_2022_3843_MOESM2_ESM.docx]

**Supplement 2. Search Strategies**

**MEDLINE (Ovid)**

| Search number | Query |
| --- | --- |
| 1 | depression/ or exp Depressive Disorder/ or Dysthymic Disorder/ or Mood Disorders/ or Affective Symptoms/ or Adjustment Disorders/ |
| 2 | (Depress* or mental health or mental disorder* or psychiatric disorder* or mood disorder* or bipolar disorder*).mp. [mp=title, abstract, heading word, drug trade name, original title, device manufacturer, drug manufacturer, device trade name, keyword, floating subheading word, candidate term word] |
| 3 | 1 or 2 |
| 4 | Transcranial Magnetic Stimulation/ OR Transcranial Direct Current Stimulation/ |
| 5 | (non invasive brain stimulation or transcranial direct current stimulation or tDCS or transcranial magnetic stimulation or TMS or repetitive transcranial magnetic stimulation or rTMS).mp. |
| 6 | 4 or 5 |
| 7 | (rct or quasi-experimental or randomi?ed controlled trial* or randomi?ed trial* or randomi?ed clinical trial* or randomi?ed control trial* or wait-list or experiment* or randomly or randomi?ed clinical trial or clinical trial* or controlled clinical trial*).mp. |
| 8 | 3 and 6 |
| 9 | 7 and 8 |

**EMBASE (via Ovid)**

| Search number | Query |
| --- | --- |
| 1 | depression/ or Mood Disorder/ or Affective Symptoms/ or Adjustment Disorders/ |
| 2 | (Depress* or mental health or mental disorder* or psychiatric disorder* or mood disorder* or bipolar disorder*).mp. [mp=title, abstract, heading word, drug trade name, original title, device manufacturer, drug manufacturer, device trade name, keyword, floating subheading word, candidate term word] |
| 3 | 1 or 2 |
| 4 | Transcranial Magnetic Stimulation/ OR Transcranial Direct Current Stimulation/ |
| 5 | (non invasive brain stimulation or transcranial direct current stimulation or tDCS or transcranial magnetic stimulation or TMS or repetitive transcranial magnetic stimulation or rTMS).mp. |
| 6 | 4 or 5 |
| 7 | (rct or quasi-experimental or randomi?ed controlled trial* or randomi?ed trial* or randomi?ed clinical trial* or randomi?ed control trial* or wait-list or experiment* or randomly or randomi?ed clinical trial or clinical trial* or controlled clinical trial*).mp. |
| 8 | 3 and 6 |
| 9 | 7 and 8 |

**PsycInfo (EBSCOhost)**

| Search number | Query |
| --- | --- |
| S1 | (DE "Transcranial Magnetic Stimulation" OR DE "Transcranial Direct Current Stimulation") |
| S2 | "non invasive brain stimulation" OR "transcranial direct current stimulation" OR tDCS OR "transcranial magnetic stimulation" OR TMS OR "repetitive transcranial magnetic stimulation" OR rTMS |
| S3 | S1 OR S2 |
| S4 | (DE "Major Depression" OR DE "Anaclitic Depression" OR DE "Dysthymic Disorder" OR DE "Endogenous Depression" OR DE "Late Life Depression" OR DE "Postpartum Depression" OR DE "Reactive Depression" OR DE "Recurrent Depression" OR DE "Treatment Resistant Depression") |
| S5 | Depress* OR "mental health" OR "mental disorder*" OR "psychiatric disorder*" OR "mood disorder*" OR "bipolar disorder*" |
| S6 | S4 OR S5 |
| S7 | (TI rct OR "quasi experimental" OR quasi-experimental OR "randomi#ed controlled trial*" OR "randomi#ed trial*" OR "randomi#ed clinical trial*" OR "randomi#ed control trial*" OR wait-list OR experiment* OR randomly OR "randomi#ed clinical trial" OR "clinical trial*" OR "controlled clinical trial*") |
| S8 | (AB rct OR "quasi experimental" OR quasi-experimental OR "randomi#ed controlled trial*" OR "randomi#ed trial*" OR "randomi#ed clinical trial*" OR "randomi#ed control trial*" OR wait-list OR experiment* OR randomly OR "randomi#ed clinical trial" OR "clinical trial*" OR "controlled clinical trial*") |
| S9 | S7 OR S8 |
| S10 | S3 AND S6 |
| S11 | S9 AND S10 |

**Web of Science (Core collection)**

| Search number | Query |
| --- | --- |
| #1 | TS= (Depress* or "mental health" or "mental disorder*" or "psychiatric disorder*" or "mood disorder*" or "bipolar disorder*") |
| #2 | TS= ("non invasive brain stimulation" or "transcranial direct current stimulation" or tDCS or "transcranial magnetic stimulation" or TMS or "repetitive transcranial magnetic stimulation" or rTMS) |
| #3 | TS= (rct or "quasi experimental" or quasi-experimental or "randomi$ed controlled trial*" or "randomi$ed trial*" or "randomi$ed clinical trial*" or "randomi$ed control trial*" or wait-list or experiment* or randomly or "randomi$ed clinical trial" or "clinical trial*" or "controlled clinical trial*") |
| #4 | #1 AND #2 AND #3 |

**PubMed**

| Search number | Query |
| --- | --- |
| #1 | "Transcranial Direct Current Stimulation"[Mesh] OR "Transcranial Magnetic Stimulation"[Mesh] OR “non invasive brain stimulation”[tw] OR “transcranial direct current stimulation”[tw] OR tDCS[tw] OR “transcranial magnetic stimulation”[tw] OR TMS[tw] OR “repetitive transcranial magnetic stimulation”[tw] OR rTMS[tw] |
| #2 | "depression"[Mesh] OR depress*[tw] OR dysthymi*[tw] OR distress*[tw] OR “common mental health”[tw] OR “mood disorder”[tw] OR “affective disorder”[tw] OR “affective symptom*”[tw] OR “adjustment disorder”[tw] |
| #3 | #1 AND #2 |
| #4 | rct[tw] OR quasi-experimental[tw] OR "randomized controlled trial*"[tw] OR "randomized trial*"[tw] OR “controlled clinical trial*”[tw] OR “wait-list”[tw] OR “experiment”[tw] OR “randomized clinical trial”[tw] OR “clinical trial*”[tw] OR “controlled clinical trial*”[tw] |
| #5 | #3 AND #4 |
