# Supplementary material for: Non-invasive brain stimulation combined with psychosocial intervention for depression: a systematic review and meta-analysis
Source: BMC Psychiatry. 2022 Apr 19;22:273. doi: 10.1186/s12888-022-03843-0 (PMC9016381; doi:10.1186/s12888-022-03843-0)
Supplement: Supplementary file 3 — Additional file 3.. [file 12888_2022_3843_MOESM3_ESM.docx]

**Supplement 3. The definition of depressive diagnostic tools of included studies.**

**Hamilton Depression Rating Score, 21-items (HDRS-21):**

0-7 score are considered as being normal

8-16 score suggest mild depression

17-23 score moderate depression

> 24 score are indicative of severe depression

**Hamilton Rating Scale for Depression (HAM-D):**

Not depressed: 0-7 score

Mild (subthreshold): 8-13 score

Moderate (mild): 14-18 score

Severe (moderate): 19-22 score

Very severe (severe): >23 score

**Montgomery-Asberg Depression Rating Scale (MADSR):**

0 to 6 score: normal/symptom absent

7 to 19 score: mild depression

20 to 34 score: moderate depression

> 34 score: severe depression

**Beck Depression Inventory (BDI):**

0-9 score: indicates minimal depression

10-18 score: indicates mild depression

19-29 score: indicates moderate depression

30-63 score: indicates severe depression

**Beck Depression Inventory-II (BDI-II):**

0-13 score: minimal depression

14-19 score: mild depression

20-28 score: moderate depression

29-63 score: severe depression

**Self-rating Depression Scale (SDS):**

20-44 score: normal range

45-59 score: mildly depressed

60-69 score: moderately depressed

> 70 score: severely depressed
