# Supplementary material for: Non-invasive brain stimulation combined with psychosocial intervention for depression: a systematic review and meta-analysis
Source: BMC Psychiatry. 2022 Apr 19;22:273. doi: 10.1186/s12888-022-03843-0 (PMC9016381; doi:10.1186/s12888-022-03843-0)
Supplement: Supplementary file 4 — Additional file 4.. [file 12888_2022_3843_MOESM4_ESM.docx]

**Supplement 4. Protocols of the NIBS and psychosocial intervention.**

| **Author,**  **year** | **Intervention group**  **(IG)** | **Control group**  **(CG)** | **Treatment sequence** | **Protocol** |
| --- | --- | --- | --- | --- |
| **tDCS Excitatory** |  |  |  |  |
| Brunoni et al., 2014 | Active tDCS  +cognitive control therapy | Sham tDCS  +cognitive control therapy | Cognitive control therapy was delivered in the last 15 min of each tDCS session. | 10 sessions, daily for 2 weeks (5 consecutive weekdays, with 2 days off during weekends), 30 min tDCS and three 5-min cognitive control therapy  Sham tDCS: Consisted of an initial 30s ramp-in phase, 30s of active stimulation and a ramp-out phase of 15s  Stimulation site:  The anode: The left DLPFC over F3.  The cathode: The right DLPFC over F4.  Stimulation parameters: 2.0 mA |
| Khayyer et al., 2018 | Active tDCS  +positive psychotherapy | CG1: Active tDCS  CG2: Positive psychotherapy | Participants received tDCS treatment followed by positive psychotherapy | 12 sessions, 3 times per week for 4 weeks, 15 min tDCS and 30 min positive psychotherapy  Stimulation site:  The anode: The left DLPFC over F3/F4 based on the EEG.  The cathode: Fz or extra cephalic.  Stimulation parameters: 1.5 mA |
| Lagueux et al., 2018 | Active tDCS  +graded motor imagery | Sham tDCS  +graded motor imagery | tDCS simultaneously with graded motor imagery | 14 sessions, daily for the first two weeks (5 consecutive weekdays, with 2 days off during weekends), and once per week (on Mondays) for the end of 4 weeks, 20 min tDCS and 3 times per day, 6 times a week for 6 weeks, 10 min graded motor imagery.  Sham tDCS: current only delivered during the first 30s.  Stimulation site:  The anode: C3 or C4 based on the 10-20 EEG system;  The cathode: The opposite supraorbital area (i.e. ipsilateral to the affected limb).  Stimulation parameters: 2.0 mA |
| Manenti et al., 2018 | Active tDCS  +computerized cognitive training | Sham tDCS  +computerized cognitive training | tDCS started from the beginning of each computerized cognitive training session | 10 sessions, daily for 2 weeks (5 consecutive weekdays, with 2 days off during weekends), 25 min tDCS and 25 min computerized cognitive training  Sham tDCS: current only delivered during the first 10s and the last 10s.  Stimulation site:  The anode: The left DLPFC over F3 based on the 10-20 EEG international system.  The cathode: The right supraorbital area.  Stimulation parameters: 2.0 mA |
| Manenti et al., 2016 | Active tDCS  +physical therapy | Sham tDCS  +physical therapy | tDCS was engaging in physical therapy | 10 sessions, 5 sessions weekly for 2 weeks, 25 min tDCS and 25 min physical therapy.  Sham tDCS: the current was turned off 10s after the beginning of the stimulation and turned on for the last 10s of the stimulation periods.  Stimulation site:  The anode: The left or right DLPFC at about halfway between F3/4 based on 10/20 EEG system.  The cathode: The contralateral supraorbital area at about halfway between F7/8.  Stimulation parameters: 2.0 mA |
| Martin et al., 2019 | Active tDCS  +cognitive training | Sham tDCS  +cognitive training | Cognitive training commenced 5 min after the onset of tDCS | 15 sessions, 3 times per week for 5 weeks, 30 min tDCS and 45-60 min cognitive training.  Sham tDCS: the current was gradually increased to 1 mA over 30s and then left on for 30 s before being ramped down over another 30s.  Stimulation site:  The anode: The left DLPFC over F3 based on the 10-20 EEG system.  The cathode: F8.  Stimulation parameters: 2.0 mA |
| Mendonca et al., 2016 | Active tDCS  +aerobic exercise | CG1: Aerobic exercise  CG2: Active tDCS | N/A | 5 sessions, daily for the first week, 20 min tDCS and/or 30 min aerobic exercise.  Stimulation site:  The anode: The primary motor cortex (M1) per the International 10/20 system at point C3 (M1 left).  The cathode: The supraorbital region, contralateral to the anode (right).  Stimulation parameters: 2.0 mA |
| Nasiri et al., 2020 | Active tDCS+  unified protocol | CG1: Unified protocol-alone  CG2: Wait-list | tDCS treatment occurred during the final two weeks of unified protocol treatment. | 10 sessions, daily for 2 weeks (5 consecutive weekdays, with 2 days off during weekends), 30 min tDCS and/or a maximum of 12, 60 min unified protocol sessions.  Stimulation site: the cathode was placed the right DLPFC; the anode was placed over contralateral deltoids.  Stimulation parameters: 2.0 mA |
| Nord et al., 2019 | Active tDCS  +cognitive behavioral therapy | Sham tDCS  +cognitive behavioral therapy | Cognitive behavioral therapy immediately following each tDCS session. | 8 sessions, weekly for 8 weeks, 20 min tDCS and 1-hour cognitive behavioral therapy (sessions occurred a minimum of 6 days apart).  Sham tDCS: current only delivered during the first 30s.  Stimulation site:  The anode: The left DLPFC over F3 based on the 10-20 EEG international system.  The cathode was placed on ipsilateral deltoid.  Stimulation parameters: 1.0 mA |
| Riberto et al., 2011 | Active tDCS  +multidisciplinary rehabilitation program | Sham tDCS  +multidisciplinary rehabilitation program | tDCS was performed before the first hour of multidisciplinary rehabilitation program. | 10 sessions, weekly for 10 weeks, 20 min tDCS and 36 sessions, 3 times a week for 4 months multidisciplinary rehabilitation program.  Sham tDCS: current only delivered during the first 30s.  Multidisciplinary rehabilitation program: educative interventions or cognitive behavior group therapy during the first hour. Cardiovascular and strengthening training or stretching exercises during the second hour.  Stimulation site:  The anode: The left DLPFC over C3 based on the 10-20 EEG international system.  The cathode: Contralateral supraorbital area.  Stimulation parameters: 1.0 mA |
| Segrave et al., 2014 | Active tDCS  +cognitive control training (CCT) | CG1: Sham tDCS  +cognitive control training  CG2: tDCS  +sham cognitive control training | Cognitive control training immediately following each tDCS session. | 5 sessions, weekly for 5 consecutive weeks, 24 min tDCS and 22 min cognitive control training.  Sham tDCS: current only delivered during the first 2 min.  Stimulation site: the anode was placed the left DLPFC over F3 based on the 10-20 EEG international system; the cathode was placed the right DLPFC over F8.  Stimulation parameters: 2.0 mA |
| Van Noppen et al., 2020 | Active tDCS  +behavioral therapy | Sham tDCS  +behavioral therapy | N/A | 15 sessions, 5 times per week for 3 weeks, 20 min tDCS and 3 sessions, 1 time per week for 3 weeks behavioral therapy  Sham tDCS: current only delivered during the first 30s.  Stimulation site:  The anode: The left DLPFC over F3 based on the 10-20 EEG international system.  The cathode: The right DLPFC over F8.  Stimulation parameters: 2.0 mA |
| Vanderhasselt et al., 2015 | Active tDCS  +neurocognitive training | Sham tDCS  +neurocognitive training | During the last 15 min of the tDCS stimulation, patients performed the neurocognitive training. | 10 sessions, 5 sessions per week for 2 weeks, 30 min tDCS and 15 min neurocognitive training.  Sham tDCS: current only delivered during the first 30s and the last 15s.  Stimulation site:  The anode: The left DLPFC over F3 based on the 10-20 EEG international system.  The cathode: The right DLPFC over F4.  Stimulation parameters: 2.0 mA |
| **rTMS**  **Excitatory** |  |  |  |  |
| Guinot et al., 2021 | Active rTMS  +exercise training | Sham rTMS  +exercise training | MT program following rTMS | 5 sessions a week for first two weeks, 2 sessions on week 3, then 1 session a week on 4, 6, 9 and 13 weeks, 20 min rTMS and 3 sessions a week (from week 3 to 14).  Exercise training: Each session started with 45 min aerobic training on an ergocycle and then 45 mins of pool-based exercises followed by 45 mins of relaxation.  Sham rTMS: Carried out with a sham coil.  Stimulation site: M1 (dominant thenar area)  Stimulation parameters: 10Hz, 80%RMT with 2000 impulsions each session |
| Li et al., 2021 | rTMS  +occupational therapy | Occupational therapy | N/A | 1 session daily, 5 sessions weekly for a total of 8 weeks, 20 min rTMS and occupational therapy.  Stimulation site: N/A  Stimulation parameters:20 Hz and 100% intensity with stimulation period of 5 seconds, and stimulation interval of 15s. |
| **rTMS Inhibitory** |  |  |  |  |
| Lee & Kim, 2018 | Active rTMS  +neurodevelopmental therapy | Sham rTMS +neurodevelopmental therapy | rTMS commenced after the onset of neurodevelopmental therapy | 10 sessions, daily for 2 weeks (5 consecutive weekdays, with 2 days off during weekends) rTMS and neurodevelopmental therapy.  Sham rTMS: Carried out with a sham coil.  Stimulation site: The right DLPFC over F4 based on the 10-20 EEG international system.  Stimulation parameters:1Hz, 110%RMT with each session consists of 50 trains of 40 pulses on each train separated by 25-second pauses. |
| Sharma et al., 2020 | Active rTMS  +conventional physical therapy | Sham rTMS  +conventional physical therapy | rTMS sessions on each day was followed by conventional physical therapy | 10 sessions, daily for 2 weeks (5 consecutive weekdays, with 2 days off during weekends), 30 min rTMS and 45 min conventional physical therapy.  Sham rTMS: N/A  Stimulation site: Fc3/Fc4 based on the 10-20 EEG international system.  Stimulation parameters:1Hz, 110%RMT, total 750 pulses with intertrain interval of 45 seconds |
